# Supplementary material for: Towards unified AI-driven fracture mechanics: the extended deep energy method (XDEM)
Source: Nat Commun. 2026 Aug 15;17:8492. doi: 10.1038/s41467-026-76748-1 (PMC13484471; doi:10.1038/s41467-026-76748-1)
Supplement: Supplementary file 3 [file 41467_2026_76748_MOESM3_ESM.zip › pic/Results/Crack_propagation/Straight/l_0.015/model/centers_step_1.pdf]

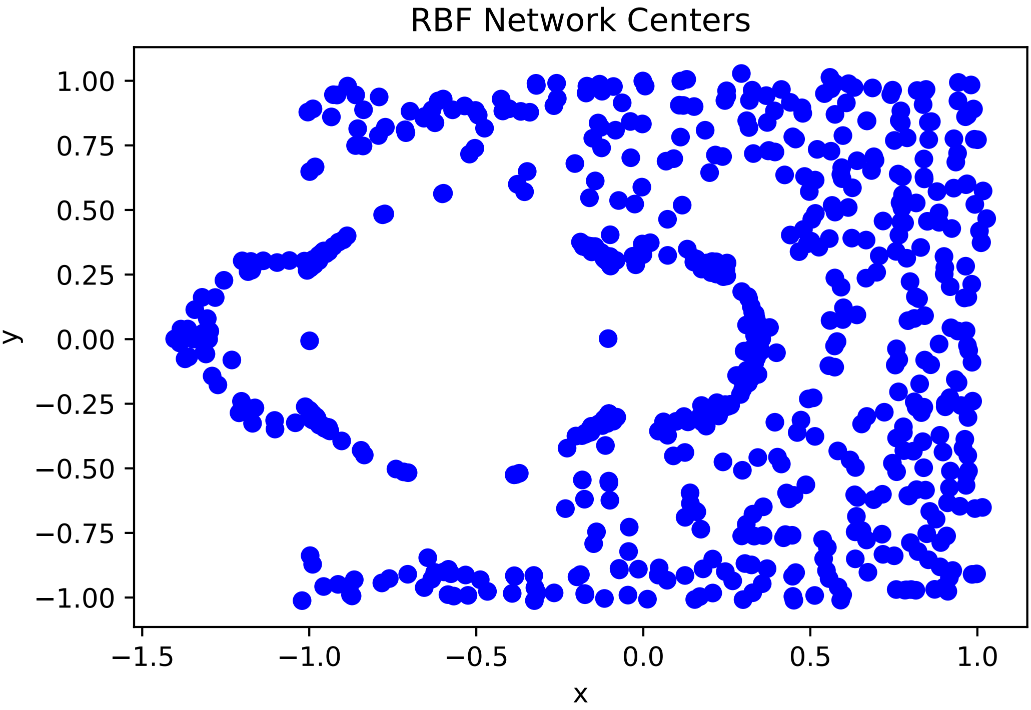
RBF Network Centers
y
1.00
0.75
0.50
0.25
0.00
−0.25
−0.50
−0.75
−1.00
x
−1.5
−1.0
−0.5
0.0
0.5
1.0
